# Using a structured decision analysis to evaluate bald eagle vital signs monitoring in Southwest Alaska National Parks

**DOI:** 10.1002/ece3.6499

**Published:** 2020-07-15

**Authors:** Rebecca Kolstrom, Tammy L. Wilson, Larry M. Gigliotti

**Affiliations:** ^1^ Department of Natural Resource Management South Dakota State University Brookings SD USA; ^2^ Southwest Alaska Network National Park Service Anchorage AK USA; ^3^ South Dakota Cooperative Fish and Wildlife Research Unit South Dakota State University U.S. Geological Survey Brookings SD USA

**Keywords:** bald eagle, long‐term monitoring, Southwest Alaska, structured decision, vital signs

## Abstract

1. Monitoring programs can benefit from an adaptive monitoring approach, where key decisions about why, where, what, and how to monitor are revisited periodically in order to ensure programmatic relevancy.
2. The National Park Service (NPS) monitors status and trends of vital signs to evaluate compliance with the NPS mission. Although abundant, The Southwest Alaska Network (SWAN) monitors bald eagles because of their inherent importance to park visitors and role as an important ecological indicator. Our goal is to identify an optimal monitoring program that may be standardized among participating parks.
3. We gathered an expert panel of scientists and managers, and implemented a Delphi Process to gather information about the bald eagle monitoring program. Panelists generated a list of means objectives for the monitoring program: minimizing cost, minimizing effort, maximizing the ability to detect change in bald eagle populations, and maximizing the amount of accurate information collected about bald eagles.
4. We used a swing‐weighting technique to assign importance to each objective. Collecting accurate information about bald eagles was considered the most important means objective.
5. Combining panelist‐generated information with objective importance, we analyzed the scenarios and defined the optimal decision using linear value modeling. Through our analysis, we found that a “Comprehensive” monitoring scenario, comprised of all feasible monitoring metrics, is the optimal monitoring scenario. Even with greatly increased cost, the Comprehensive monitoring scenario remains the best solution.
6. We suggest further exploration of the cost and effort required for the Comprehensive scenario, to determine whether it is in the parks’ best interest to begin monitoring additional metrics.

## Graphical abstract

The Southwest Alaska Network (SWAN) of the National Park Service monitors bald eagles as part of the Vital Signs Monitoring Plan. This study uses structured decision‐making techniques to evaluate an optimal long‐term bald eagle monitoring program that may be standardized among participating parks.

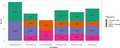

## 1 INTRODUCTION

The collection of long‐term datasets, termed monitoring, is an important part of ecosystem science, management, and conservation world‐wide (Janetos & Kenney, 2015). Following the “roadmap” by Reynolds, Knutson, Newman, Silverman, and Thompson (2016) for designing and implementing a monitoring program, an adequate program includes steps to encompass the general phases of framing the problem, designing the monitoring program, implementing and learning, and learning and revising. This type of monitoring fits into the scope of “adaptive monitoring,” which is motivated by specifying objectives and answering clear questions through long‐term monitoring. In this adaptive monitoring framework, all decisions about monitoring should be iterative (Lindenmayer & Likens, 2009), as values and attitudes may change over the course of an extended period of time (Williams, 2011). Repeatedly revisiting decisions related to monitoring data collection allows a monitoring program to remain relevant with changing agency priorities (Oakley, Thomas, & Fancy, 2003). Instead, many programs begin by collecting data before laying the groundwork, and the value of the monitoring effort may be diminished (Reynolds et al., 2016). A structured approach to decisions about a monitoring protocol ultimately leads to a more efficient program by identifying the optimal survey design for monitoring (Reynolds, Thompson, & Russell, 2011).

Structured decision‐making is defined by Gregory et al. (2012) as “the collaborative and facilitated application of multiple objective decision‐making and group deliberation methods to environmental management and public policy problems.” It can be compared to and fit into an adaptive framework, as both exhibit the similarities of defining explicit objectives and alternatives. Structured decision‐making approaches can serve as decision aids to facilitate monitoring programs that explicitly address the decisions about protocols or implementation, and can help to conserve limited resources by reducing the waste of time and effort (Gregory et al., 2012; Lyons, Runge, Laskowski, & Kendall, 2008; Neckles, Lyons, Guntenspergen, Shriver, & Adamowics, 2015). Ultimately, monitoring programs that spend an adequate amount of time defining objectives and optimizing the program based on factors that are important to the decision‐makers are more successful as their monitoring is focused on important data needs for conservation and wildlife issues (Nichols & Williams, 2006; Oakley et al., 2003). Ideally, structured decision‐making is best enacted at the conception of a monitoring program, but can be used to review or revise a monitoring program as needed.

Long‐term monitoring programs are collaborative in nature, involving multiple agencies and decision‐makers. Although it may be easier to shy away from decisions involving multiple decision‐makers, acknowledging the opinions of multiple experts can encourage deeper thinking from individuals (Runge, Converse, & Lyons, 2011). Additionally, a structured process may allow multiple decision‐makers to better understand the specifics and reasoning behind alternatives and may foster consensus among a decision team (Mattson et al. 2019, Thorne et al., 2015). Unfortunately, collaborative decisions about monitoring objectives tend to be hindered by logistical constraints (i.e., cost) and a desire to maintain existing survey methods, which can prevent improvements in monitoring (Reynolds et al., 2016). Furthermore, there are often multiple objectives, such as social ideals, and the value of collecting scientific information (Grimble & Wellard, 1997), that may be important to consider when considering a monitoring protocol. A monitoring decision that makes explicit trade‐offs to meet all objectives collectively will enable the data to be put to its best use (Lyons et al., 2008; Nichols & Williams, 2006). It is recommended that an open discourse be created and upheld between field scientists, managers, those analyzing the data, and other stakeholders throughout the decision‐making process to maintain support for decisions regarding the monitoring protocol (Reynolds et al., 2011). By highlighting trade‐offs, the cost (not just monetarily) of choosing one alternative over another can be examined (Grimble & Wellard, 1997).

For the National Park Service (NPS), vital signs monitoring enacted by the inventory and monitoring division (IMD) is intended to evaluate the health of ecosystems in order to measure the ability of NPS to uphold its mission “…To conserve the scenery and the natural and historic objects and the wild life therein and to provide for the enjoyment of the same in such manner and by such means as will leave them unimpaired for the enjoyment of future generations” (Fancy, Gross, & Carter, 2009). Each network was set up in an adaptive monitoring framework based on conceptual models of ecosystem function relevant to each of the 32 monitoring networks. Individual vital signs were selected by each network so that they provided information necessary to learn about system dynamics depicted by the conceptual models.

In the process of creating a bald eagle monitoring program for the Southwest Alaska Network (SWAN), decision‐makers did not fully explore key portions of framing the problem and designing objectives (Reynolds et al., 2016). As a result, the parks currently collect data on bald eagles slightly differently from one another and are not able to use their data as effectively as possible. In this paper, we present a case study that uses structured decision‐making to inform a decision about the future of the long‐term bald eagle monitoring program in Southwest Alaska National Parks. By using structured decision‐making tools to identify monitoring metrics used for the long‐term bald eagle monitoring program in the Southwest Alaska Network of National Parks, we will review programmatic goals and examine the trade‐offs of monitoring scenarios made up of different monitoring metrics of interest for managers. It should be noted that while parks in the Southwest Alaska Inventory and Monitoring Network monitor bald eagles as part of the Vital Signs Monitoring Plan, bald eagles are not actively managed in the parks, making this a case study of using structured decision‐making techniques in an adaptive monitoring framework to evaluate a long‐term status and trends monitoring program.

Means objectives focus on the manner in which a more basic goal, or fundamental objective, can be achieved (Gregory et al., 2012). In this decision context, all defined objectives are means objectives to the fundamental objective of optimizing the long‐term bald eagle monitoring program for Southwest Alaska National Parks. A multi‐agency panel of scientists and managers has already defined means objectives and a suite of potential monitoring metrics to use when evaluating the monitoring decision though a Delphi Process (Kolstrom, Wilson, & Gigliotti, 2020; Linstone & Turoff, 2002). These means objectives were quantified using responses from the Delphi questionnaires (Kolstrom et al., 2020). Now, by considering the means objectives, we identify the optimal decision about monitoring metrics that can be used in the long‐term monitoring program by using a linear value modeling approach.

Our main objective is to identify a set of monitoring metrics that is expected to maximize the efficiency of monitoring, while balancing the means objectives of minimizing cost, minimizing effort, maximizing accurate information collected, and maximizing the ability to detect change. Experts chose to base the decision on these four factors because these adequately represent the benefits of and limitations to the long‐term bald eagle monitoring program for this particular National Park network.

We developed a decision model, which we used to evaluate the sensitivity of our decision to changes in objective weights. We also explored sensitivity of the optimal decision to experimental increases in cost. We used our model to make suggestions to the Southwest Alaska Inventory and Monitoring Network about how to standardize the long‐term bald eagle monitoring program across the five participating parks The methods we have chosen to select an optimal bald eagle monitoring program provide an example case study that uses structured decision‐making techniques to formally and transparently analyze complex problems and make a decision that combines the opinions of many experts.

## 2 METHODS

### 2.1 Study area

Bald eagles are abundant in Alaska, with populations in the state estimated around 30,000 (Alaska Department of Fish & Game, 2017). Southwest Alaska provides suitable coastal habitat for bald eagles, many of which reside on National Park Service land in this area (Wilson, Weiss, Shepherd, Phillips, & Mangipane, 2017). The Southwest Alaska Inventory and Monitoring Network (SWAN) is comprised of five units of the National Park Service, including coastal parks Katmai National Park and Preserve (KATM), Kenai Fjords National Park (KEFJ), and Lake Clark National Park and Preserve (LACL) (Bennett, Thompson, & Mortenson, 2006; National Park Service, 2018a). Along with Wrangell‐St. Elias National Park and Preserve (WRST), which is part of the Central Alaska Inventory and Monitoring Network (CAKN), these parks are home to large populations of breeding bald eagles (National Park Service, 2018b; Wilson et al., 2017). Bald eagles in the parks are monitored annually by SWAN and CAKN as part of their Vital Signs Monitoring Plan (Bennett et al., 2006; Wilson et al., 2017).

### 2.2 PrOACT: Forming the decision context and analyzing the decision problem

Methods for this process were based around the PrOACT concept: Problems, Objectives, Alternatives, Consequences, Trade‐offs (Hammond 2015). This study was conceived to address the problem of how to best standardize long‐term bald eagle monitoring in Southwest Alaska National Parks. Objectives were defined by a panel of experts, which included decision‐makers. Alternatives consist of realistic monitoring scenarios for this study system. Consequences were first examined among monitoring metrics to narrow down an extensive list of metrics to a more manageable list of feasible metrics. Consequences of competing objectives were then examined through a swing‐weighting process of the selected objectives. Finally, trade‐offs were examined through a linear value model that calculates a utility value for each monitoring scenario. Methods are described in more detail, below.

We convened an expert panel of 17 scientists, managers, and personnel from the National Park Service, US Fish and Wildlife Service, and South Dakota Game, Fish, & Parks to participate in a Delphi Process, where we identified important stressors for bald eagles in Alaska and linked stressors to monitoring metrics (Kolstrom et al., 2020; Linstone & Turoff, 2002). We compiled this expert panel using a snowball process. We selected scientists from all participating parks and other experts who expressed interest in participating in the process. We asked these panel members to suggest other members to be included in the expert panel until we received no more suggestions.

We queried the panel about long‐term bald eagle monitoring in Southwest Alaska National Parks and gathered information about the cost, effort, reliability, and sensitivity of monitoring metrics commonly used to monitor bald eagle populations (Kolstrom et al., 2020). Through an in‐person panel meeting, we formed means objectives for bald eagle monitoring program decisions in Southwest Alaska Network (SWAN) parks: minimize cost, minimize effort, maximize ability to detect changes in bald eagle populations, and maximize accurate information about bald eagles. The expert panel chose to separate the objectives regarding cost and effort to ensure that staff time was being considered appropriately. Separating these two objectives allowed staff time to be considered as a necessary resource, beyond the cost of paying for the fieldwork (e.g., aircraft contracts). This was meant to ensure that the time of salaried employees (whose salaries will not change, regardless of the effort required of a monitoring program) will be considered in the decision as a resource being used. The objective to maximize ability to detect changes in bald eagle populations emphasized the panelists’ desire to measure metrics that will indicate changes in bald eagle populations in the parks quickly enough to respond with management action. By assigning an objective of maximizing accurate information, panelists hoped to increase knowledge about bald eagles and bald eagle populations in the parks.

We then evaluated the consequences of individual monitoring metrics based on the four means objectives. A comprehensive list of monitoring metrics was formed through structured expert elicitation, the Delphi Process, that uses surveys to combine expert opinion derived from a panel of Federal managers and eagle experts (Kolstrom et al., 2020). Using information collected through the Delphi Process and a consequence table, the comprehensive list of metrics was narrowed to the six best‐performing metrics based on cost, effort, reliability, and sensitivity. The monitoring metrics that remained in consideration after this process are as follows: total number of bald eagle nests, changes in distribution, productivity, proportion of nests used by bald eagles for reproduction, total number of nesting pairs, and adult survival. Methods used to obtain this list of six metrics are described in more detail in Kolstrom et al. (2020).

To form alternative monitoring scenarios, we used combinations of the six best‐performing monitoring metrics. Although there are many alternative scenarios that can be formed using subsets of the six selected metrics, we chose six scenarios to represent monitoring options that were considered feasible by a park scientist (Table 1). The scenario “Status Quo” included feasible metrics that are currently monitored by the parks during three flight surveys. Two of these surveys investigate nest initiation and the third investigates nest productivity. The “Comprehensive” scenario consisted of all six metrics determined to be feasible by the expert panel. There is also a scenario, “No Monitoring” that considered the option to discontinue monitoring bald eagles. “New Metrics” considers metrics that are feasible, but not currently monitored by the parks (adult survival and changes in distribution). There are also two scenarios “Reduced Status Quo 1” and “Reduced Status Quo 2” that considered some of the currently monitored metrics with a reduced monitoring effort that results in lower estimator precision and could introduce bias. The Reduced Status Quo 1 scenario reduced sampling during the second nest initiation survey. Rather than revisiting all previously surveyed nests, a 50% random sample of nests would be revisited. The Reduced Status Quo 2 scenario would completely remove the second nest initiation survey, but would increase effort of the productivity survey to include all nests found in the first survey. We designed these scenarios to cover a range of reasonable options that are comprised of the feasible metrics identified by the experts.

**Table 1:** Metrics included in each monitoring scenario considered in the decision about long‐term bald eagle monitoring program for SWAN parks

	Comprehensive	Status Quo	Reduced Status Quo 1	Reduced Status Quo 2	New Metrics	No Monitoring
Total number of bald eagle nests	X	X	X	X		
Productivity	X	X	X*^rp^*	X*^rp^*		
Proportion of nests used by bald eagles for reproduction	X	X	X*^rp^*	X*^rp, ub^*		
Total number of nesting pairs	X	X	X*^rp^*	X*^rp, ub^*		
Changes in distribution	X				X	
Adult survival	X				X	

Note

Scenarios include metrics considered feasible by the expert panel. Reduced Status Quo 1 and Reduced Status Quo 2 incorporate the same metrics as the Status Quo scenario, but remove various amounts of survey effort. *rp* designates that a metric is measured with reduced precision. *ub* designates a metric that is measured with unquantifiable bias.

The methods we used to evaluate and rank alternatives are based on the Simple Multi‐Attribute Rating Technique (Edwards, 1971, 1977). We scored scenarios based on the means objectives for the bald eagle monitoring program (minimize cost, minimize effort, maximize accurate information about bald eagles, and maximize ability to detect changes in bald eagle populations). For each objective, we used expert panelist responses from the Delphi process to quantify scores for cost, effort, reliability, and sensitivity.

We asked panelists to assign a cost value to each metric, for each annual year of surveying in one park. We gave multiple choice options for each metric: $0–5,000; $5,000–10,000; $10,000–15,000; $15,000–20,000; $20,000–25,000; and $25,000+. Panelist responses to the multiple choice question were combined into a weighted average value.

To calculate an effort score for each scenario, we asked experts to estimate annual person days required for each individual metric and calculated the mean across panelists for each metric. For each scenario, we summed the mean annual effort values for individual metrics that comprise the scenario.

As a measure of the amount of accurate information collected about bald eagles, we asked panelists to assign a reliability score to each monitoring metric. This was based on the premise that more reliable metrics will increase the amount of accurate information collected. A reliability score was generated through the use of multiple‐choice questions administered to panelists. Scores were based on experts’ evaluations of the amount of accurate information about bald eagles provided by each metric. Panelists rated the reliability of metrics on a 5‐point scale, and we calculated the weighted average for each metric; this weighted average represents the reliability score. We added these values for the metrics that comprise each scenario to create a reliability score for each scenario.

The ability to detect change was measured using a sensitivity score. To create this sensitivity score, experts were asked to select metrics that are responsive to important stressors to bald eagles. The sensitivity score for each metric is a count of the stressors to which that metric is responsive. For each scenario, we added the sensitivity scores of the metrics that comprise the scenario. By linking monitoring metrics to important stressors, we were asking panelists to indirectly evaluate how sensitive each monitoring scenario is to important changes in the system. By framing the survey questions in this manner, we were also able to craft a conceptual model of the system.

For the two reduced effort Status Quo scenarios, we did not collect information about the means objectives directly from the expert panel. Aided by a park scientist, we assigned values to these scenarios based on their relative performance to the Status Quo scenario. Since the Status Quo scenario is comprised of three annual flight surveys, we estimated that one third of each score is attributed with each annual survey. Using these approximations and current park budgets, we calculated scores for scenarios by eliminating 50% of the second initiation flight from one annual survey (Reduced SQ1) or by eliminating the second initiation flight from the annual survey and adding 50% to the productivity survey (Reduced SQ2). We normalized values for each scenario on a 0–1 scale, and those normalized values are used in the decision model (Table 2).

**Table 2:** Normalized scores representing predicted outcomes for means objectives under alternative strategies for monitoring bald eagles in Southwest Alaska National Parks

Scenario	Cost	Effort	Detect change	Accurate information
Status Quo	0.402	0.513	0.515	0.659
Comprehensive	0	0	1	1
No Monitoring	1	1	0	0
New Metrics	0.598	0.487	0.485	0.341
Reduced SQ1	0.519	0.594	0.429	0.549
Reduced SQ2	0.635	0.675	0.343	0.439

Note

Cost and effort values are calculated by subtracting the normalized score from 1, since these objectives are being minimized.

We determined the weight of means objectives based on importance. These weights were determined by the panel of experts using a swing‐weighting technique, adapted from Gregory et al. (2012). All panel members who were willing to participate in this task completed a swing‐weighting form. We distributed a form to each panelist using Google Sheets. In this Google Sheet, we listed each means objective along with corresponding performance metrics, and whether our aim is to maximize or minimize that attribute. We displayed a range of values, including the worst and best possible values for each attribute. The worst and best values are generated from the range of score responses from the Delphi Process questionnaires. We also displayed five hypothetical situations. A “Benchmark” situation is comprised of the worst possible values for all four means objective attributes. In the remaining four hypothetical situations, all attributes were set to their worst values except for one attribute in each situation, which was set to its best value.

We asked panelists to rank the four hypothetical situations from 1 to 4 (1 is best). The Benchmark situation was automatically assigned the worst rank of 5. By doing this, we were asking the panelists which attribute they would swing to its best level, if they could only pick one. That situation received the rank of 1. The next most important swing was ranked 2, etc. We then asked panelists to score each situation based on its priority. The Rank 1 situation automatically received a score of 100. Panelists assigned scores in decreasing amounts to the remaining hypothetical situations based on importance in achieving each measure swing. We provided the example to panelists that if they score their Rank 2 situation at 50, they are insinuating that it is half as important to achieve that measure swing as the measure swing in their Rank 1 situation, which has a score of 100. Using Equation 1, we assigned a weight to each means objective for each individual panelist and created box and whisker plots for each objective.

(1) $$\text{weight}\left(\text{normalized}\right)=\left[\frac{\text{score}}{\text{sum of scores}}\right]\times 100$$

To combine panelist responses for cumulative objective weights that will be used in the decision model, we averaged individual panelist *weight (normalized)* values for each means objective (Equation 1).

To examine trade‐offs of each monitoring scenario, we then combined normalized scenario scores and means objective weights to create our decision model. This decision model uses a technique called linear value modeling, also known as linear additive modeling. A utility score is calculated for each monitoring scenario by multiplying that scenario's score for a particular objective by that objective's weight. The products are then added for all objectives to create the utility score for each scenario, as demonstrated by the following equation: Utility = Σ*W_i_X_i_*, where *W_i_* is the weight of means objective *I*, and *X_i_* is the performance score for each means objective i. (Gregory et al., 2012).

**FIGURE 1:**
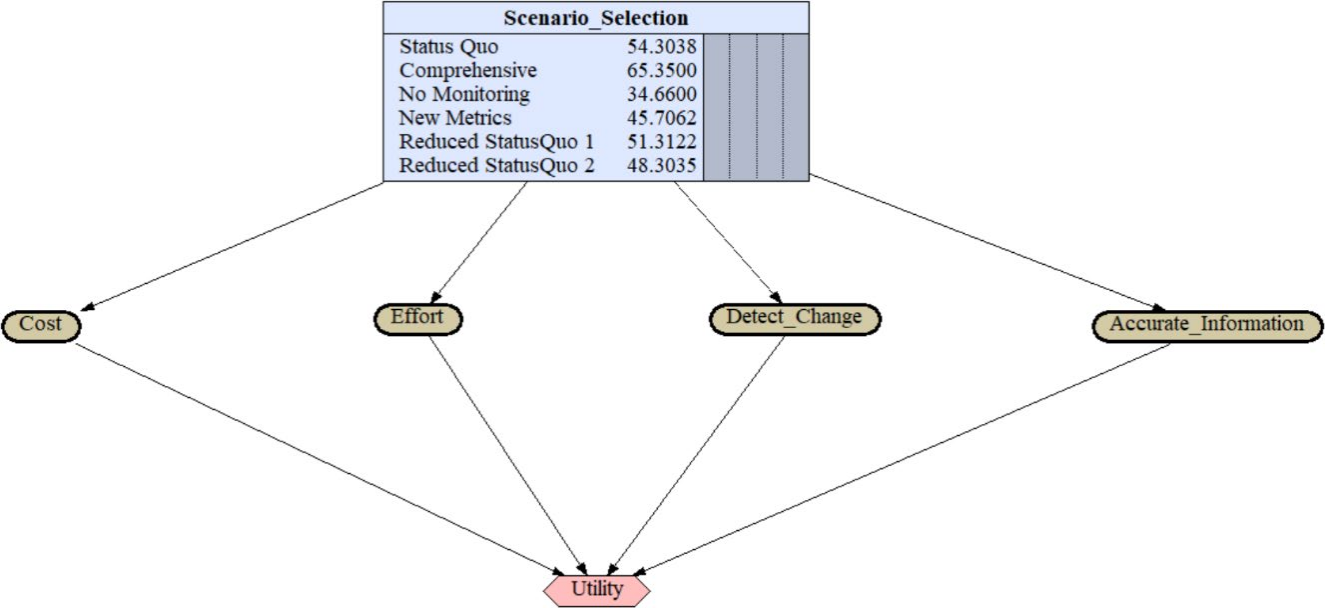
A diagram using a linear value modeling equation to combine scenario scores and means objective weights into a utility score. Scenarios for long‐term bald eagle monitoring in Southwest Alaska National Parks are displayed with their utility scored in the Scenario_Selection box. The boxes for each means objective are compiled with scenario scores for each objective. The utility box combines the data and provides the score in the Scenario_Selection box. The Comprehensive monitoring scenario has the highest utility value, making it the optimal decision

We displayed the decision model using program Netica from Norsys Software Corp. The decision net uses three types of nodes: a decision node, nature nodes, and a utility node. The decision node allows the user to select a scenario alternative and displays the utility value of each scenario. The decision node connects to four nature nodes, which correspond to each means objective. These nature nodes are thus named “Cost,” “Effort,” “Accurate_Info,” and “Detect_Change.” Using the normalized score values for each objective, we populated the model in Netica. Since this model is not probabilistic in nature, we did not assign distribution to nature nodes. Rather, we used program Netica to provide a visual representation of the decision model. These values are then routed through the utility node to calculate the utility score. The value model incorporated the weight assigned to each objective by expert panelists, using the swing‐weighting technique. These weights may be changed to examine the effect that changing values may have on the decision outcome. Our linear value model used (*1—normalized value*) for “Cost” and “Effort” since our goal is to minimize these attributes. We used the normalized values for “Accurate_Info” and “Detect_Change” since our goal is to maximize these attributes. The utility values are displayed in the decision node (Figure 1). The scenario with the highest utility value is considered the optimal decision.

We performed a sensitivity analysis to determine the change in objective weights needed to alter the outcome of the decision model. To examine sensitivity, we graphed the percent total utility for each monitoring scenario across objective weights ranging from 0 to 100. Percent total utility is a measure of an individual scenario's utility score compared to the utility scores of all scenarios combined at a particular objective weight. By examining intersections in the sensitivity graphs, we determined at which weight one scenario began to outcompete another.

**FIGURE 2:**
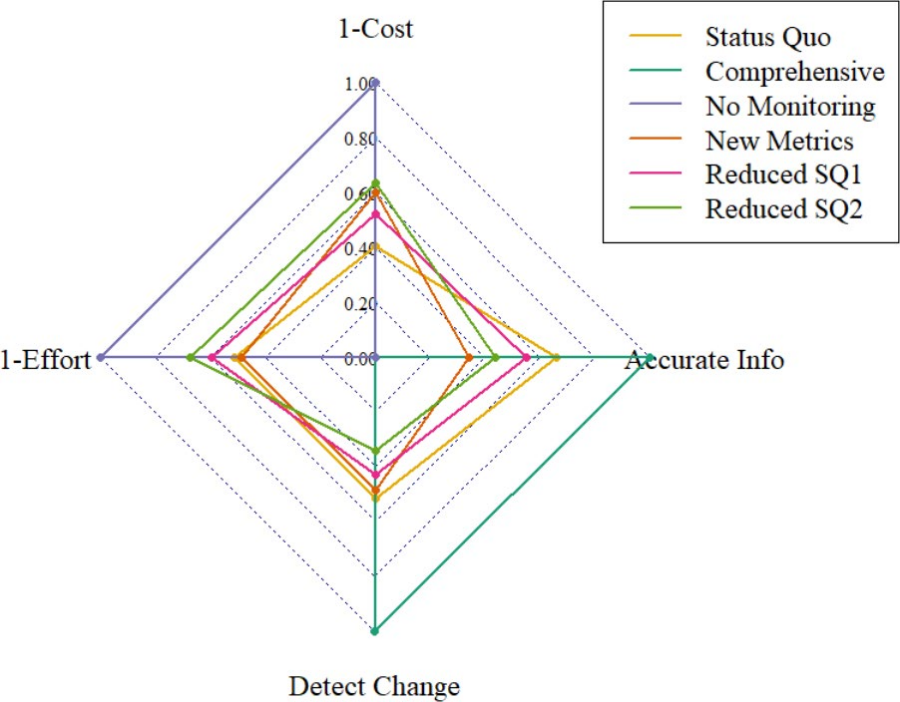
Normalized scores for each means objective defined in the decision about bald eagle monitoring in Southwest Alaska National Parks. For the cost and effort objectives, this chart displays 1‐normalized values so that higher scores on this chart represent better‐performing scenarios for each objective. Points farther from the origin on each axis are considered “better‐performing” with regard to that axis's means objective

As we began to analyze our results, we noted that the manner in which our survey measured costs created a maximum cost of “$25,000+.” This combined with further research about realistic costs of measuring adult survival and concerns that this metric was underestimated in cost led us to provisionally test increased cost values for the measures of this metric. These increased cost values were presented to our expert panel during an in‐person meeting, where we confirmed that experimentally increasing costs were verified (Figure 2).

By experimentally increasing the cost of the Comprehensive monitoring scenario, we examined the sensitivity of the decision to cost. We explored how much more expensive the Comprehensive scenario must be to no longer outcompete the Status Quo scenario. We tested various increased cost values for the Comprehensive scenario, to the point where the cost of the Comprehensive scenario is 500 times more expensive than the Status Quo scenario. We compared this to the proportional Utility value of the Comprehensive monitoring scenario to the Status Quo monitoring scenario.

## 3 RESULTS

None of the monitoring scenarios outperformed all other monitoring scenarios for all four means objectives, illustrating the trade‐offs inherent in the monitoring decision problem (Figure 2). The No Monitoring scenario performed best regarding the means objectives of minimizing cost and minimizing effort. The Comprehensive scenario performed best regarding the means objectives of maximizing the amount of accurate information and maximizing the ability to detect change. Behind the Comprehensive scenario, the Status Quo scenario was the next best‐performing scenario regarding the objectives of maximizing the amount of accurate information and maximizing the ability to detect change.

**FIGURE 3:**
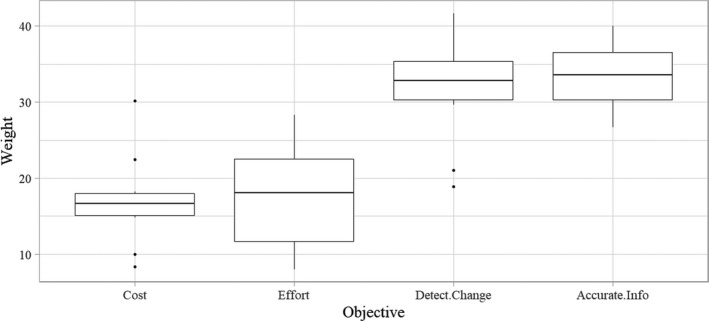
Boxplots showing the distribution of panelist weights (*n* = 10) for the four means objectives of the long‐term bald eagle monitoring program in SWAN. These weights were collected through a swing‐weighting procedure, and average weights are used in the final decision model. A boxplot is shown for each objective, and the median is displayed on each plot. Outliers are defined as any points that lie beyond the distance from the hinge to 1.5 * (Interquartile Range). Outliers are represented by dots. The range of each boxplot represents the range of individual panelist responses

Panelists ranked the means objectives of ability to detect change and accurate information about bald eagles higher than cost and effort objectives (Figure 3). Among panelists, cost weights ranged from 8.3% to 30.2%. Effort weights ranged from 8% to 28.3%. Detect Change weights ranged from 18.9% to 41.7%. Accurate Info weights ranged from 26.7% to 40%. Mean weights were calculated to be used in the decision model. Accurate Info had the highest mean weight (33.1%), followed by Detect Change (32.3%), Effort (17.6%), and Cost (17.1%).

**FIGURE 4:**
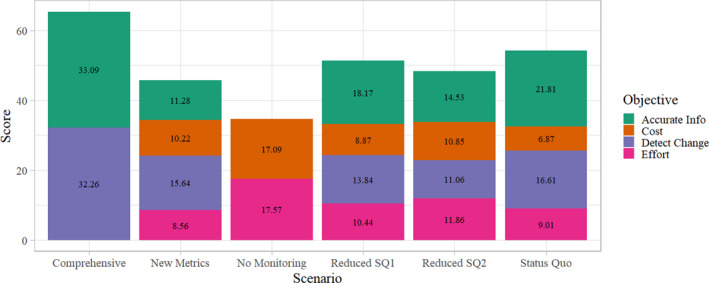
Expected utilities of potential long‐term bald eagle monitoring strategies in SWAN by objective. The scores displayed in each portion of the bars were calculated by multiplying the scenario's score for that means objective by the means objective weight. Objective scores for each scenario add to that scenario's total utility score

The linear value model calculated the overall utility score for each scenario by combining objective scores and means objective weights. The Comprehensive monitoring scenario was identified as optimal by the model. The Comprehensive monitoring scenario has a utility score of 65.35. It is followed by the Status Quo scenario (54.30), Reduced Status Quo 1 (51.31), Reduced Status Quo 2 (48.30), New Metrics (45.71), and No Monitoring (34.66). Based on scores and objective weights, No Monitoring was the least desirable monitoring scenario (Figure 4). The best‐performing solution, the Comprehensive scenario, according to this model, was comprised of scores from Detecting Change and collecting Accurate Information. Although it received scores of zero for cost and effort, it still outranked all other scenarios.

**FIGURE 5:**
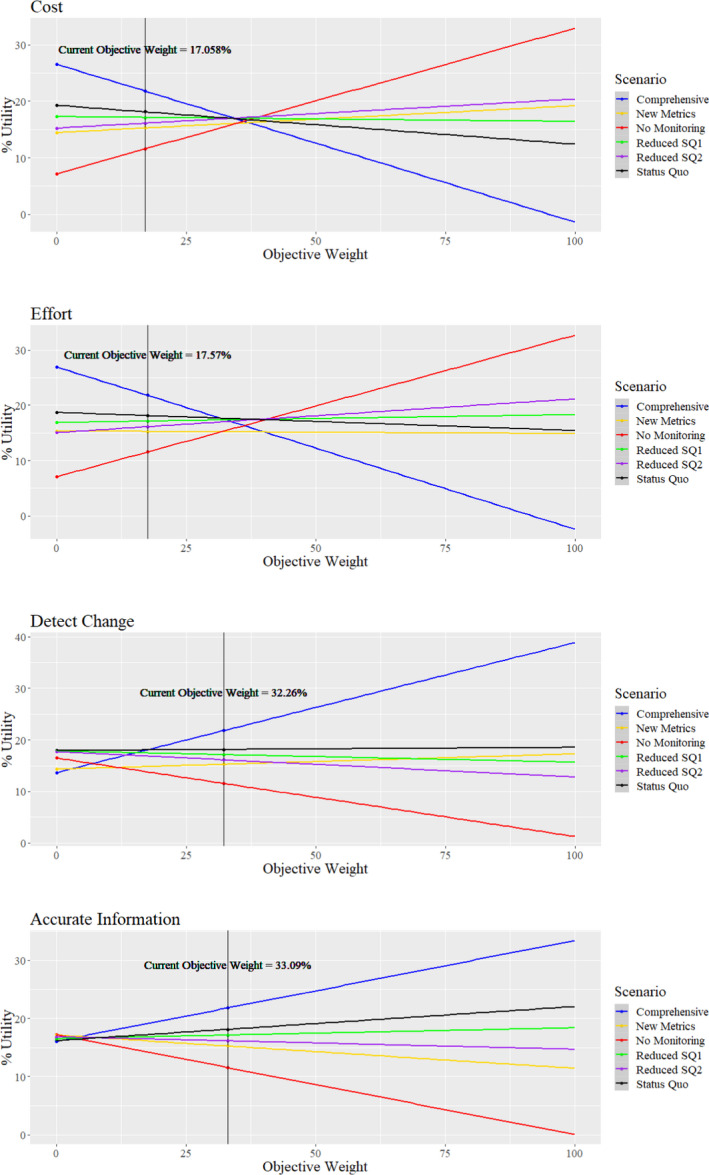
Response of % utility to changes in each objective weight for the decision about long‐term bald eagle monitoring in SWAN. Current objective weights are noted. The optimal scenario at a particular objective weight is designated by the order of the scenario lines, descending vertically. At current objective weights, the scenarios in descending order from optimal are as follows: (1) Comprehensive; (2) Status Quo; (3) Reduced SQ1; (4) Reduced SQ2; (5) New Metrics; and (6) No Monitoring

The sensitivity analysis revealed the point at which changing objective weights would change the optimal decision (Figure 5). For most objective weights, either the Comprehensive monitoring scenario or No Monitoring scenario was ranked highest. At very low objective weights for cost, the Comprehensive monitoring scenario performed the best of all scenarios. Once the value of cost increased to a weight of 34.4%, intermediate scenarios were most optimal, until cost was valued at a weight of 37.9%, when No Monitoring became most optimal. Similarly, if effort was valued at low objective weights, the Comprehensive monitoring scenario outcompeted all other scenarios, until it reached an objective weight of 31.7%. At that point, the Status Quo scenario outcompeted all other scenarios, and fell below Reduced Status Quo 2 for a very narrow window, until No Monitoring began to outcompete all other monitoring scenarios at an objective weight of 40.8%.

There were few changes in scenario rankings when varying Accurate Information and Detect Change objectives. These objective weights must decrease to low values for the Comprehensive scenario to be outcompeted by another monitoring scenario. All monitoring scenarios had similar utilities until Accurate Info reached an objective weight of 4.7%, when the Comprehensive scenario quickly outcompeted all other scenarios. When varying the objective weight for Detect Change, the Status Quo scenario outcompeted all other metrics until the objective weight reached 17.3%, when the Comprehensive scenario became the top‐ranked scenario.

**FIGURE 6:**
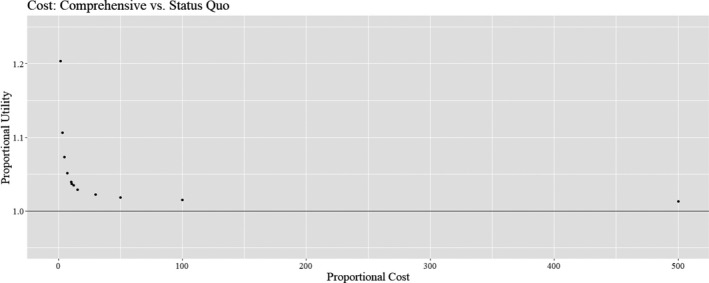
Effect of increasing the Proportional Cost (*Cost of Comprehensive Scenario/Cost of Status Quo Scenario*) on the Proportional Utility (*Utility of Comprehensive Scenario/Cost of Comprehensive Scenario*). A change in the optimal monitoring decision would be indicated by a point that falls below the threshold of *Proportional Utility = 1*

Experimentally increasing the cost of the Comprehensive survey to 500 times the value of the Status Quo scenario did not change the optimal decision. This is represented by comparing the proportional utility of the Comprehensive and Status Quo scenarios to the proportional cost of these scenarios (Figure 6).

## 4 DISCUSSION

An “adaptive monitoring” approach should be based on clearly defined questions and should adopt an iterative approach to developing these questions, collecting data, and interpreting data (Lindenmayer & Likens, 2009). Successful monitoring programs should also place focus on initial planning and collaborative learning (Reynolds et al., 2016). In this decision context of long‐term bald eagle monitoring in Southwest Alaska National Parks, we queried a team of decision‐makers about the study system and the long‐term monitoring program to provide an initial analysis of the decision problem. By doing so, we have set the stage continue making better monitoring decisions in an adaptive monitoring context.

Structured decision‐making adds rigor and reflection to decisions about scientific monitoring programs. In the decision about long‐term bald eagle monitoring in Southwest Alaska National Parks, it allowed decision‐makers with differing viewpoints to better understand the value and intention of the monitoring program. We quantitatively compared several monitoring scenarios for bald eagles in four national parks in Alaska, based on means objectives defined by relevant experts. Although it requires the maximum amount of cost and effort of any scenario option, our decision model identifies the Comprehensive scenario as the optimal monitoring program. This includes monitoring for all feasible metrics to gather the most information about bald eagles as possible, and maximize the ability to detect changes in the population. A structured approach to the decision about long‐term bald eagle monitoring for this park system may help park managers to consider alternatives that may not have otherwise been considered, due to the risk of losing consistency by changing a monitoring program from what it has always been. By using a structured decision model, we illustrate the importance of carefully evaluating the resources required of a project before changing the monitoring protocol.

Although any number of monitoring scenarios could have been analyzed using these methods, we chose six scenarios that we felt adequately represented the range of options that vary in the cost and effort they require as well as the information they provide. Following guidelines from Gregory et al. (2012), the alternatives that are considered when making a decision should be able to provide a complete and meaningful resolution to the problem at hand. We chose not to include all possible combinations of monitoring metrics as monitoring scenarios, as some of these would have been unreasonable. For example, some metrics such as “total number of bald eagle nests” can be measured concurrently while surveying additional metrics. Therefore, eliminating this metric would not reduce cost or effort. In addition to the obvious combinations of currently monitored and newly proposed metrics, we explored two scenarios that monitored current metrics with reduced cost and effort. Although these scenarios would still provide some information about currently monitored metrics, they would introduce noise and, in the case of Reduced SQ2, bias. This would affect our ability to detect changes in the bald eagle population. We calculated the cost and effort for these reduced scenarios based on a previous budget, and the values entered for amount of accurate information and ability to detect change were estimated with the help of an expert. While we feel these values adequately represent these reduced scenarios, we suggest that if these are options the Southwest Alaska Network chooses to pursue in the future, they thoroughly examine the costs and effort days required at that time. However, we do suggest that these options are not pursued, as the Status Quo monitoring program outcompeted reduced programs at a wide range of objective weights.

Competing means objectives make it necessary to consider trade‐offs, which are inevitable in natural resource decisions (Converse, Moore, Folk, & Runge, 2013; Gregory et al., 2012). Our decision model helped to quantify those trade‐offs and identified the Comprehensive monitoring scenario as the optimal decision. Given the weight of the means objectives, the increased ability of this scenario to generate accurate information about bald eagles and detect changes in the population greatly outweighed the negative trade‐offs of high cost and effort. The Status Quo scenario scored relatively well and outcompeted most other scenarios. However, the relatively high accurate information scores given by the panel to the adult survival metric propelled the Comprehensive scenario to outweigh the Status Quo scenario under a wide range of objective weights. Both Reduced Status Quo scenarios were eliminated due to the reduction in accurate information and ability to detect change, despite a reduced cost and effort, which had relatively low importance to the expert panelists. Monitoring just the newly suggested metrics, adult survival and changes in distribution, would provide a significant amount of information and ability to detect change. However, the cost and effort are not low enough for this scenario to outweigh the current monitoring regime. No Monitoring was eliminated since it would provide no information about bald eagles and provide no ability to detect changes in the populations. Without bald eagle monitoring, if bald eagle populations began to decline, parks may be too late to identify the decline and intervene with management action.

The results of our decision model relied on the weights assigned by panelists. Since cost and effort were both valued at relatively low weights, the calculated expected utility of each scenario was largely based on its ability to generate accurate information about bald eagles and detect changes in populations without substantial regard to overall program cost. In fact, the monitoring scenario that ultimately had the largest expected utility was the worst performer in terms of cost and effort (it received scores of 0 for these objectives), but performed well enough in its ability to generate accurate information and detect change that it outcompeted all other monitoring scenarios. As Gende, Hendrix, and Schmidt (2018) explore the balance between resources and park values while managing human visitors, our decision balances the resources needed to conduct a monitoring program and the values of park scientists. Primarily, these values consist of the information collected from the bald eagle monitoring program. In the case of our decision model, the balance between resources and information leans heavily toward the data collected on bald eagles, thus determining the outcome of the decision model.

It became apparent while examining the means objective weights that a simplified, two‐objective decision model adequately exemplifies the decision context. Although panelists specified four means objectives of minimizing cost, minimizing effort, maximizing the amount of accurate information collected, and maximizing the ability to detect changes in the population, there is a clear distinction between two sets of objective weights. Cost and effort were weighted very similarly, as were detecting change and collecting accurate information. As the discussion of an optimal monitoring program continues, it may be beneficial to reduce the decision to a more simplistic cost‐benefit analysis, with cost and effort combined into a “resources required” objective and accurate information and detecting change combined into an “information obtained” objective. While simplifying the problem to two means objectives would still be considered a multi‐criteria decision analysis, the decision can be improved with greater simplicity (Mendoza & Martins, 2006).

Sensitivity analyses that vary objective weights point to only narrow ranges of objective weights that allow intermediate scenarios to outcompete the more extreme scenarios. At many objective weights of cost and effort, this decision about long‐term monitoring can be considered “all or nothing,” with either the Comprehensive or No Monitoring scenarios outcompeting all other monitoring scenarios. In our decision model, there would need to be a dramatic shift in values for another monitoring scenario to outcompete the Comprehensive scenario (either much more importance placed on cost and effort, or less value assigned to accurate information and detecting change).

The scores assigned to each objective and used as inputs in the decision models were generated using expert opinion, with the panel of experts including decision‐makers. This is a valid method of collecting information to supplement empirical data (Eycott, Marzano, & Watts, 2011; MacMillan & Marshall, 2006) and evaluating the desired outcomes involved in a monitoring decision (Gregory et al., 2012). Expert panel selection can influence model outcome (Krueger, Page, Hubacek, Smith, & Hiscock, 2012), and although we used a snowball sampling technique to identify experts, we were limited to individuals whom we knew, and who had time to participate. It is also important to allow flexibility in level of participation among stakeholders during a decision‐making process (Mattson et al. 2019). As a result, our panel included primarily biologists, as opposed to higher‐level managers. Likely, a panel of biologists will place a higher value on the persistence of a species and knowledge about population trends than the cost and effort it requires.

Future work should include conducting the objective‐weighting exercise with a panel of directors and budgetary decision‐makers to see how the weights of objectives changes. We suggest that this be explored before making any changes to the bald eagle monitoring program. However, the higher‐level managers who participated on our panel deferred to the biologists to score the information value of proposed metrics. The extremely high information value of the metric adult survival would be difficult to overcome with higher utility weights for cost and effort. When presenting our results to the expert panel, we acknowledged the higher importance placed on the information collected. One panelist suggested that as decisions continue to be discussed regarding the long‐term bald eagle monitoring program, the weighting process could be presented to higher‐level managers and the decision model could be revised accordingly.

While we addressed uncertainty across panelists by performing sensitivity analyses, future improvements to this process could address the uncertainty in individual panelist response. Asking panelists to estimate parameters within high and low bounds, with a reasonable level of confidence, would allow us to create probability distributions to describe uncertainty (Martin, Runge, Flewelling, Deutsch, & Landsberg, 2017). With additional information, the constructed linear value model could be populated with elicited probabilities. Additionally, calculating the expected value of perfect information (EVPI) would help to strengthen our sensitivity analysis (Runge et al., 2011). As more information is obtained, the decision framework should be re‐evaluated and changes should be expected (Neckles et al., 2015).

Additionally, a limitation of our study was ambiguity surrounding the monitoring metric “Adult Survival.” Since survival is not currently monitored, we asked panelists to make educated guesses about the values that were entered in the model. However, since methods to measure adult survival were not specified, panelists were likely considering differing methods when estimating performance measures. As an example, one panelist suggested measuring survival by collecting feathers from tree bases, and thus had lower estimates of cost and effort. An additional limitation was the way in which the question about cost was presented (panelists chose cost from a list of options, the greatest of which was> $25,000). This limited the cost of monitoring adult survival to a value that was likely much less than the realistic cost. As this is an initial analysis of the decision problem, we suggest future iterations of this decision analysis explore a more thorough analysis into the exact needs of the parks to monitor adult survival and changes in distribution. A more specific cost estimate to monitor these metrics should be generated, as well as a more specific statement of how this information will be used. By doing so, decision‐makers can further analyze the trade‐offs involved in taking on this more intensive monitoring effort.

Low response rates to questions about estimating cost and effort of various monitoring metrics limited our response data. Some panelists felt unqualified to make those estimates, and these questions were asked late in the Delphi Process when response rates were lower. Porter, Whitcomb, and Weitzer (2004) found that longer surveys usually correlate with lower response rates. Since, for some measures of cost and effort, we had as few as three respondents estimating values of performance measures, these values may not be as accurate as other estimates. Estimates of cost and effort varied greatly. For future expert elicitation, a structured protocol may help to ensure the information collected is as accurate as possible (Hemming, Walshe, Hanea, Fidler, & Burgman, 2018).

To mitigate the variation associated with the cost of the adult survival metric, we evaluated the effects of increasing survey cost on the model outcome. The decision model was not sensitive to increasing costs. It is worth noting that any change in monitoring program will require additional resources in the form of cost and effort to write new protocols, train staff, create new data templates, and analyze and interpret data. Thus, the decision model most likely does not accurately represent the value of cost to decision‐makers.

This process displayed the inherent importance of gathering accurate information and being able to detect changes in populations of symbolic and charismatic wildlife populations to decision‐makers, as quantified by the high value placed on these two means objectives. Our decision model identified the Comprehensive monitoring scenario consisting of all feasible monitoring metrics as the best scenario. Even in relatively undisturbed wildlife populations, such as the bald eagle populations in Southwest Alaska National Parks, park scientists are driven by the need to maintain an adequate bank of information about the species and its health. As an important ecological indicator, the bald eagle can provide knowledge about the health of the park ecosystems. Although there is an expressed desire to minimize the resources required to monitor bald eagles in the parks: Katmai National Park and Preserve, Kenai Fjords National Park, Lake Clark National Park and Preserve, and Wrangell‐St. Elias National Park and Preserve, the structured decision‐making process highlighted the relatively low value of cost and effort to decision‐makers when determining the optimal monitoring program.

This process provides an example of using structured decision techniques to inform practical conservation decisions by addressing a unique decision problem about long‐term monitoring without associated management action. As future information is collected and priorities of decision‐makers may change, iterative analysis of this decision problem can help to provide the basis for a successful and efficient monitoring program. We believe that examining the decision problem through a documented and structured process allowed our team of decision‐makers to focus on the specifics of the monitoring program and fostered consensus surrounding the monitoring decision.

## CONFLICT OF INTEREST

The authors declare no conflicts of interest.

## AUTHOR CONTRIBUTION

**Rebecca Kolstrom:** Data curation (equal); Formal analysis (lead); Investigation (equal); Methodology (equal); Writing‐original draft (lead); Writing‐review & editing (lead). **Tammy L Wilson:** Conceptualization (lead); Data curation (equal); Formal analysis (supporting); Funding acquisition (lead); Investigation (equal); Methodology (equal); Project administration (lead); Supervision (equal); Writing‐original draft (supporting); Writing‐review & editing (supporting). **Larry M. Gigliotti:** Conceptualization (lead); Data curation (equal); Formal analysis (supporting); Funding acquisition (lead); Investigation (equal); Methodology (equal); Project administration (lead); Writing‐original draft (supporting).

Rebecca Kolstrom wrote the majority of the content in this manuscript, wrote surveys and communicated with the Bald Eagle Expert Panel throughout the Delphi process, and analyzed the results of the process. Rebecca Kolstrom serves as the corresponding author on this manuscript. Dr. Tammy Wilson and Dr. Larry Gigliotti conceived this project and provided guidance throughout all stages of this process. Dr. Wilson provided knowledge about bald eagle monitoring in the SWAN system, contributed substantially to the content of the Delphi Process, contributed to the writing and editing of this manuscript, and oversaw research and ideas throughout the entirety of the process. Dr. Larry Gigliotti provided guidance as an expert in Human Dimensions, helped to facilitate the Delphi Process with the Bald Eagle Expert Panel, assisted with editing the content in this manuscript, and oversaw research and ideas throughout the entirety of the process.

## Data Availability

Data are free and available to the public through the National Park Service Integrated Resource Management Applications (IRMA) Data Store. https://irma.nps.gov/DataStore/Reference/Profile/2274232
